# Ribophorin II promotes cell proliferation, migration, and invasion in esophageal cancer cells *in vitro* and *in vivo*

**DOI:** 10.1042/BSR20182448

**Published:** 2019-05-07

**Authors:** Yongshun Li, Changrong Huang, Qizhou Bai, Jun Yu

**Affiliations:** Department of the first thoracic surgery, Gansu Provincial people’s Hospital, Lanzhou 730000, China

**Keywords:** cell proliferation, cell migration, esophageal cancer, invasion, RPN2

## Abstract

Esophageal cancer is a common digestive tract cancer, which is a serious threat to human health. Ribophorin II (RPN2) is a part of an N-oligosaccharyltransferase complex, which is excessively expressed in many kinds of cancers. In the present study, we explore the biological role of RNP2 in esophageal cancer. First, we found that the expression of RPN2 was higher in esophageal cancer tissues than in adjacent non-tumor tissues, and negatively correlated with E-cadherin expression. RPN2 expression levels in esophageal cancer tissues were positively associated with differentiation and tumor node metastasis (TNM) stage. Furthermore, the expression of RPN2 was increased significantly in esophageal cancer cell lines compared with normal cells. The effect of RPN2 down-regulation on cell proliferation, cell migration, and cell invasion was examined by cell counting kit-8 (CCK8), wound healing assay, and Transwell assay, respectively. Silencing RPN2 effectively inhibited cell proliferation of esophageal cancer cells *in vitro* and *in vivo*. Cell migration and invasion were also weakened dramatically by siRPN2 treatment of esophageal cancer cells. In addition, protein expression of proliferating cell nuclear antigen (PCNA), matrix metalloproteinase (MMP-2), and E-cadherin in esophageal cancer cells was determined by Western blot analysis. PCNA, MMP-2, E-cadherin, Snail and phosphorylation-Smad2/3 expression was also regulated notably by siRPN2 treatment. These findings indicate that RPN2 exhibits oncogenetic capabilities in esophageal cancer, which could provide novel insights into esophageal cancer prevention and treatment.

## Introduction

Esophageal cancer is the sixth-leading cancer-related cause of death in the world, and about thirty thousand people worldwide die of esophageal cancer every year [1]. It is also the main malignant cancer of the upper digestive tract found in the Chinese population. Because early signs of esophageal cancer are asymptomatic, many patients are diagnosed at advanced stages, and the 5-year survival rate is only 15% [2,3]. Early diagnosis and treatment can significantly improve the survival rate [4,5]. Simple and convenient screening methods can enable early diagnosis of esophageal cancer, and will greatly improve the prognosis and reduce mortality [6,7]. At present, early diagnosis by molecular biological methods in order to improve esophageal cancer prognosis has become an important direction of medical research.

Ribophorin II (RPN2) is a part of an N-oligosaccharyl transferase complex, and RPNI and II are two types of ribophorines [8]. Previous studies reported that the expression of RPN2 is increased in breast cancer [9], gastric cancer [10], colon cancer [11], nasopharyngeal carcinoma [12], and non-small-cell lung cancer [13]. RPN2 over-expression could promote cell viability, migration, and invasion of many kinds of tumor cells. Previous study indicated that RPN2 expression predicted response to docetaxel in oesophageal squamous cell carcinoma [14], while few studies reported the biological role of Ribophorin I (RPN1) in the occurrence and progression of cancers. In addition, the underlying mechanism by which RPN1 and RPN2 regulates the progression of esophageal cancer is still not well known. From The Cancer Genome Atlas (TCGA) database (https://www.cancer.gov/types/esophageal), we found both RPN1 and RPN2 were overexpressed in esophageal cancer tissues. Moreover, RPN2 silencing showed anti-tumor effect on esophageal cancer, while RPN1 knockdown showed no significant effect on esophageal cancer cells. Thus, RPN2 was selected for further studies.

In the present study, we explore the biological role of RPN2 in esophageal cancer. First, we found abnormally increased expression of RPN2 in esophageal carcinoma tissues and cell lines. Then, the effect of RPN2 on cell proliferation, migration, and invasion was examined, and the underlying mechanism involved was studied.

## Materials and methods

### Patient samples

A total of 32 samples of esophageal cancer tissues and adjacent non-tumor tissues were surgically removed and collected in the Gansu Provincial people’s Hospital from 2013 to 2016. Preoperative clinical and pathological follow-up data were obtained from all patients. Ethical approval for the study was provided by the ethics committee of the Gansu Provincial people’s Hospital. Written informed consent from patients was obtained and complied with the Declaration of Helsinki.

### Immunohistochemistry

Tumor tissue and normal tissue were freshly isolated and fixed in 10% neutral buffered formalin and then embedded in paraffin wax. Tumor sections with a thickness of 4 μm were mounted onto slides. Slides were deparaffinized with xylene, rehydrated with ethanol, and incubated with H_2_O_2_ at 37°C for 10 min. Following blocking using 1.5% normal goat serum at 37°C for 20  min, sections were incubated overnight with antibody (Ab224347, dilution 1:200). The sections were incubated with biotin-conjugated goat-anti-rabbit immunoglobulin G secondary antibody (diluted with 3% bovine serum albumin/PBS) at 37°C for 30  min, and then incubated with horseradish peroxidase-conjugated streptavidin at 37°C for 30 min. 3, 3′-diaminobenzidine (DAB) was used as chromogenic agent. Images were obtained using a fluorescence microscope (FSX100; Olympus, Southend-on-Sea, U.K.).

### Cell culture

Human esophageal epithelial cells (Het-1A) and esophageal cancer cell lines (CP-D and CP-C) were purchased from the Chinese Academy of Sciences Cell Bank (Shanghai, China) and were cultured in RPMI1640 (Gibco) containing 10% FBS, 10 ng/ml epidermal growth factor, 1% insulin (First Biological and Chemical Medication Co., LTD, Shanghai, China), 5 μg/ml hydrocortisone (The Third Pharmaceutical Company, Beijing, China) and 1% penicillin/streptomycin at 37°C in a humidified atmosphere containing 5% CO_2_.

### siRNA transfection

CP-D and CP-C cells were seeded onto six-well culture plates at a density of 3 × 10^5^ cells/well. RPN2 siRNA and control siRNA, both purchased from Shanghai GenePharma Co., Ltd (China), were then transfected into the cells at 50–60% confluence using Lipofectamine™^2000^ (Invitrogen, Shanghai, China) following manufacturer’s protocol. After 48 h, the transfected cells were collected and processed for subsequent experiments. The sequence of siRNA used was forward: 5′-CCTAGAGGTACCGGAATGCGTTAAGCTATACCTGCATTGGCTAGTTAACGTAGAACCG-3′ and reverse: 5′-CCTACGTTAAGCAATTCAATTTTTTGTATAGCTTAACTACCGCATTTCGAGGTAGTAG-3′. The sequence of siRNA sequences of the negative siRNA used was: 5′-ACGCCUCCCGAACGUTTUUCUUGUCGUC-3′.

### Cell proliferation

Cell viability was evaluated by the cell counting kit-8 (CCK8) assay. In brief, 48 h after transfection, CP-D and CP-C cells were seeded at a density of 4 × 10^3^ cells/well in 96-well plates and incubated for 0, 24, 48, and 72 h. Subsequently, 20 μl CCK8 was added to each well and incubated for another hour. The optical density (OD) values were read at 450 nm using a microplate reader (Thermo, U.S.A.). Concentrations were measured in triplicates.

### *In vivo* xenograft experiments

Male BALB/c nude mice (6-weeks old; *n*=4) were purchased from Beijing HFK Bioscience Co. Ltd. (Beijing, China) and maintained under pathogen-free conditions with approval by the Committee of the Gansu Provincial people’s Hospital. For tumor propagation analysis, 1 × 10^7^ CP-D tumor cells transfected with short hairpin RNA targeting RPN2 (shRPN2, sequence: forward: 5′-GGAGGAGATTGAGGACCTTGT-3′ and reverse: 5′-GGATTCCAGGATGCGATTGTCG-3′) or negative control (shNC, sequence: 5′-ACGCCUCCCGAACGUTTUUCUUGUCGUC-3′) were subcutaneously injected into BALB/c nude mice. Tumor volume was calculated using the formula: volume = πab2/6 (a, tumor length; b, tumor width) at the indicated time points. Tumor weight was measured at week 5 post-injection. Animal experiments were performed in accordance with relevant guidelines and regulations of the Animal Care and Use Committees at the Gansu Provincial people’s Hospital, and a signed document issued by the Animal Care and Use Committees that granted approval was obtained.

### Histopathology

The tumor tissues were embedded in paraffin. The paraffin blocks were cut into 5-μm thick sections and mounted on glass slides. The sections were deparaffinized and rehydrated through xylene and graded alcohols. Then, the sections were stained with hematoxylin and eosin (H&E) for morphological evaluation under a light microscope.

### Wound healing assay

Cells were seeded in six-well culture plates until they formed a monolayer (100% confluence). The cell monolayer was scraped using a pipette and washed twice with medium to form a wound. The cells were further cultured in medium for 24 h and scratch closure was observed using an inverted microscope (Olympus, Tokyo, Japan). Cells were observed at 0 and 24 h after scraping, and the corresponding photographs were taken. The cell-free area at 24 h after wound forming and the original denuded area were measured using Image J software (National Institutes of Health, Bethesda, MD, U.S.A.).

### Cell invasion assay

Transwell invasion assay was performed to assess cell invasion. The upper surface of the filter (pore size, 8.0 μm; Biosciences, Heidelberg, Germany) was coated with basement membrane Matrigel (BD Bioscience) at a concentration of 2 mg/ml and incubated at 4°C for 3 h. Cells (2 × 10^4^) were seeded into the upper chamber with 200 μl serum-free medium. The lower chamber was supplemented with 750 μl medium containing 10% FBS. Following incubation for 24 h at 37°C, cells were fixed with 4% polyoxymethylene and stained with 0.5% crystal violet (Sigma–Aldrich). Then, stained cells were counted under a microscope. Five visual fields were selected and the average number was calculated.

### RNA extraction and quantitative RT-PCR

Total RNA was extracted from the patients’ tissues samples using Trizol (Invitrogen Carlsbad, CA, U.S.A.) and treated with DNase I (Roche, Indianapolis, IN, U.S.A.) to remove residual DNA according to the manufacturer’s protocol. qRT-PCR was used to examine the expression levels of RPN2 mRNA. A total of 2 μg RNA was reversely transcribed to cDNA with an oligo (dT) primer using a cDNA synthesis kit (Thermo Fisher, Rockford, IL, U.S.A.), and then used for quantitative PCR using SYBR Green qPCR Master Mixes (Thermo Fisher) according to manufacturer’s instructions. *GAPDH* mRNA levels were used for normalization. The oligonucleotides used as PCR primers were: RPN2, forward: 5′- CAAAGTCACCGGACAAGGTC-3′ and reverse: 5′-TGGTGTTCCGAAGTTGGTCA-3′ (product: 142 bp); E-cadherin, forward: 5′-AGCAGCCCCTTGTAAGC-3′ and reverse: 5′-ACTCCGTGGCATCTGTTC-3′ (product: 148 bp); PCNA forward: 5′-GCCTGACAAATGCTTGCT-3′ and reverse: 5′-GCGGGAAGGAGGAAAGT-3′ (product: 130 bp); MMP-2 forward: 5′-CGCCTTTAACTGGAGCAAA-3′ and reverse: 5′-AGGTTATCGGGGATGGC-3′ (product: 141 bp); Snail forward: 5′-GAGGCGGTGGCAGACTA-3′ and reverse: 5′-CCCCGACAAGTGACAGC-3′ (product: 143 bp); GAPDH, forward: 5′- ACCCAGAAGACTGTGGATGG-3′ and reverse: 5′- TCAGCTCAGGGATGACCTTG-3′ (product: 124 bp). The ABI 7300 system (Applied Biosystem, Foster City, CA, U.S.A.) was programmed to initially incubate the samples at 95°C for 10 min and then to denature at 95°C for 10 min. This process was followed by 40 cycles of 95°C for 15 s and 60°C for 45 s. RPN2 mRNA expression was calculated using the 2^−ΔΔ*C*^_t_ method. Data represent the average of three replicates.

### Western blot

RPN2 siRNA and control siRNA were transfected into esophageal cancer cells at a density of 5 × 10^5^ cells/well in six-well plates and, after 48 h, cells were harvested and washed twice with PBS. Cells were lysed in ice–cold radio immunoprecipitation assay buffer (RIPA, Beyotime, Shanghai, China) with freshly added 0.01% protease inhibitor PMSF (Amresco, Shanghai, China) and incubated on ice for 30 min. Cell lysates were centrifuged at 10,000 × ***g*** for 5 min at 4°C, and the supernatant (20–30 μg of protein) was run on a 10% SDS/PAGE gel and transferred electrophoretically to a nitrocellulose (NC) membrane (Millipore, Shanghai, China). Proteins were then detected with anti-RPN2 (Ab186117; dilution 1:1000), anti-E-cadherin (Ab15148; dilution 1:800), anti-matrix metalloproteinase (MMP-2) (Ab37150; dilution 1:1200), anti-proliferating cell nuclear antigen (PCNA) (Ab18197; dilution 1:800), anti-Snail (Ab53519; dilution 1:800), anti-phosphorylation-Smad2/3 (p-Smad2/3, Ab202445, dilution 1:1000), and Smad2/3 (Ab202445; dilution 1:1000). Protein loading was estimated using mouse anti-GAPDH monoclonal antibody (Cat. No. AG019 and AF006 [Beyotime, Shanghai, China]; dilution 1:2500). Proteins were visualized using enhanced chemiluminescence (ECL, Thermo Scientific, Shanghai, China).

### Statistical analysis

Results are presented as the mean ± S.D. of three independent experiments, and the data were processed with IBM SPSS13.0 software. Data for multiple comparisons were subjected to one-way ANOVA and a χ^2^ test. Spearman’s correlation analysis was used to determine the correlations between the levels of RPN2 and E-cadherin in esophageal cancer tissues. The *P*<0.05 was considered statistically significant.

## Results

### Increased RPN2 expression in esophageal cancer tissues and cell lines

**Figure 1:**
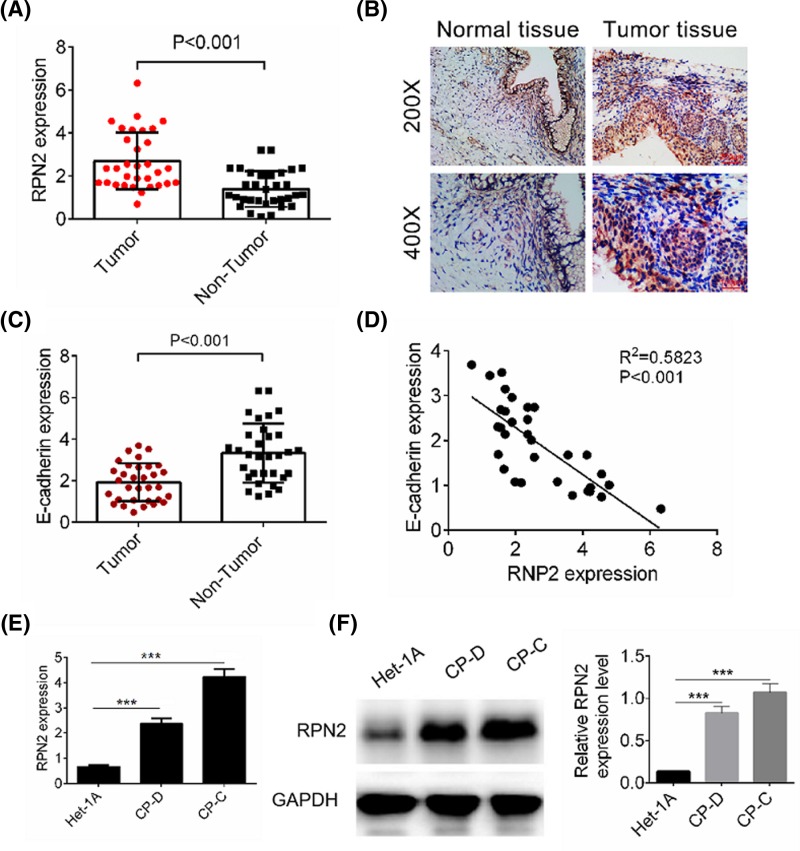
RPN2 expression in esophageal cancer tissues and cell lines (**A**) Analysis of RPN2 expression levels in 32 paired human esophageal cancer samples and non-tumor tissue by RT-qPCR. (**B**) The expression of RPN2 in tumor tissue and normal tissue was examined by IHC analysis. (**C**) Analysis of E-cadherin expression levels in 32 paired human esophageal cancer samples and non-tumor tissue by RT-qPCR. (**D**) The E-cadherin expression in esophageal cancer samples showed a negative correlation with RPN2 expression. (**E**) Expression levels of RPN2 in human esophageal epithelial cell line (Het-1A) and esophageal cancer cell lines (CP-D and CP-C) were determined by RT-qPCR. (**F**) The protein expressions of RPN2 in human esophageal epithelial cell line (Het-1A) and esophageal cancer cell lines (CP-D and CP-C) were detected by Western blot analysis. Data are expressed as the mean ± S.D. for three independent experiments. ^***^P<0.001 versus Het-1A cell.

We previously found that both RPN1 and RPN2 were increased in esophageal cancer tissues; however, RPN1 knockdown showed no significant effect on esophageal cancer cell proliferation and invasion (Supplementary Figure S1). To evaluate the function of RPN2 in esophageal cancer, we quantified RPN2 expression in esophageal cancer tissue and adjacent normal esophageal tissue by RT-PCR and Immunohistochemistry (IHC) analysis. RPN2 expression in esophageal cancer tissues was significantly increased compared with normal tissue (*P*<0.001, Figure 1A,B). The mRNA expression of E-cadherin in esophageal cancer tissues was also examined (Figure 1C), and E-cadherin expression showed a negative correlation with RPN2 expression (Figure 1D). Then, we detected the expression levels of RPN2 in a human esophageal epithelial cell line (Het-1A) and in two esophageal cancer cell lines (CP-D and CP-C) by RT-PCR and Western blot. As expected, the expression of RPN2 was increased significantly in CP-D and CP-C cells compared with Het-1A cells (*P*<0.001, Figure 1E,F). To further investigate the clinicopathological significance of RPN2 level in patients with esophageal cancer, the 30 patients were divided into two subgroups based on the mean value: low RPN2 group (19 cases) and a high RPN2 group (11 cases). As presented in Table 1, RPN2 levels in esophageal cancer tissues were positively associated with differentiation and tumor node metastasis (TNM) stage. The results indicate that RPN2 might exert tumor-promoting action in esophageal cancer.

**Table 1:** Relationship between RPN2 expression and clinicopathological features of esophageal cancer patients

Features	Number	RPN2 expression		*P*-value
		Low (*n*=19)	High (*n*=13)	
Age				
<60	16	11	5	
>60	16	8	8	0.225
Gender				
Males	17	9	8	
Females	15	10	5	0.317
Size of tumor				
<5 cm	16	12	4	
>5 cm	16	7	9	0.064
Differentiation				
Moderate-high	13	4	9	
Low	19	15	4	0.008*
Lymph node metastasis				
Positive	12	5	7	
Negative	20	14	6	0.125
TNM stage				
I–II	18	14	4	
III–IV	14	5	9	0.018*

The mean RPN2 expression level was used as the cutoff. For analysis of association between RPN2 levels and clinical features, Pearson’s χ2 tests were used.

*P<0.05.

### Down-regulation of RPN2 expression inhibited cell proliferation of esophageal cancer cells *in vitro* and *in vivo*

**Figure 2:**
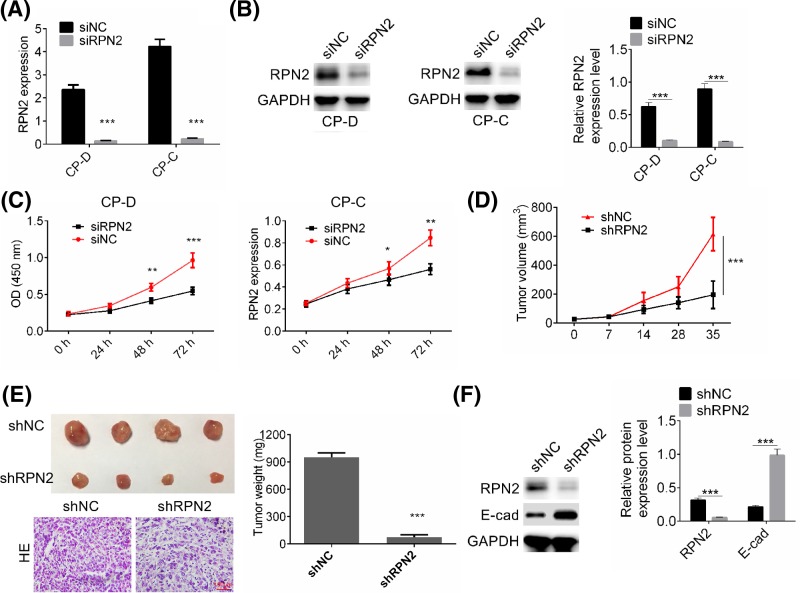
Effect of RPN2 knockdown on cell proliferation of esophageal cancer cells *in vitro* and *in vivo* (**A,B**) CP-D and CP-C cells were transfected with siRNP2 or negative control followed by RT-PCR and Western blot detection of RNP2 expression. (**C**) Cell proliferation of CP-D and CP-C cells transfected with siRNP2 or siNC was determined by CCK8 assay. (**D**) Tumor growth curves were established by detecting tumor volume every 7 days for 35 days after injection. (**E**) Tumors were isolated from nude mice in each treatment group and were weight on day 35 after injection. Tumor tissues were stained with H&E for morphological evaluation. (**F**) The protein expression of RPN2 and E-cadherin in tumors with shRPN2 or shNC transfection was identified by Western blot. GAPDH was used as a loading control. Data are expressed as the mean ± S.D. for three independent experiments. ^*^P<0.05, ^**^P<0.01, and ^***^P<0.001 versus siNC or shNC group.

In order to examine the role of RPN2 in tumor growth in esophageal cancer, CP-D and CP-C cells were transfected with siRNP2 and negative control plasmid. Transfection efficiency was examined by RT-PCR and Western blot analysis. (Figure 2A,B). Cell proliferation was then determined by CCK8 assay, and the results show that siRPN2 effectively inhibited cell viability of esophageal cancer cells 48 and 72 h after transfection compared with siNC transfected cells (*P*<0.05, Figure 2C). The tumor-suppressive effect of silencing RPN2 was also studied using *in vivo* xenograft experiments. CP-D cells transfected with shNC or shRPN2 were implanted subcutaneously into nude mice. The tumor volume of each mouse was measured every 7 days. Down-regulation of RPN2 significantly delayed tumor growth *in vivo* (*P*<0.001, Figure 2D). At 5-week post-implantation, the nude mice were killed, and tumors were photographed and weighed, and tumor tissues were stained with H&E for morphological evaluation (Figure 2E). The H&E staining showed that tumor cells in the shNC group exerted dense growth, and tumor cells in shRPN2 group showed a loose state of growth. Furthermore, RPN2 knockdown dramatically reduced the tumor size and weight (*P*<0.001, Figure 2E). The protein expression of RPN2 and E-cadherin was then identified by Western blot, and the results showed that E-cadherin expression was decreased significantly in xenografted tumors with shRPN2 transfection compared with that in shNC groups (*P*<0.001, Figure 2F). The results indicate that overexpression of RPN2 might promote cell proliferation in esophageal cancer.

### Down-regulation of RPN2 suppressed cell migration and invasion of esophageal cancer cells

**Figure 3:**
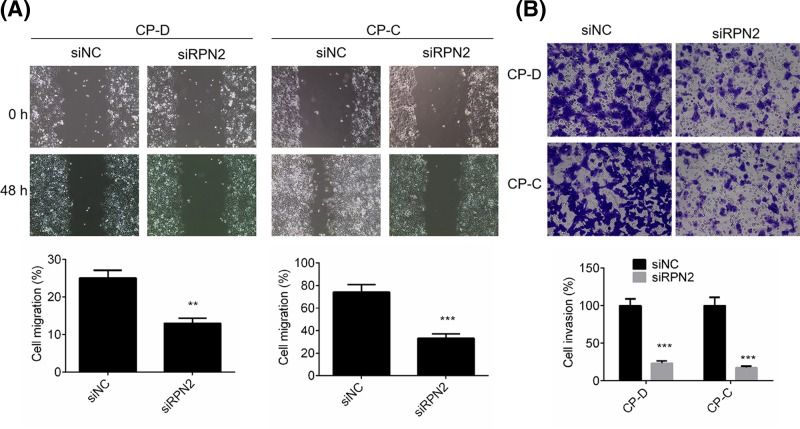
Effect of siRPN2 on cell migration and invasion of esophageal cancer cells (**A**) Cell migration of CP-D and CP-C cells transfected with siRNP2 or siNC was identified by wound healing assay. (**B**) Cell migration of CP-D and CP-C cells transfected with siRNP2 or siNC was tested by Transwell assay. Data are expressed as the mean ± S.D. for three independent experiments. ^**^P<0.01 and ^***^P<0.001 versus siNC group.

To evaluate the function of RPN2 in the progression of esophageal carcinoma, the migration and invasion of CP-D and CP-C cells transfected with siRNP2 and negative control plasmid were determined by wound healing assays and Transwell assays, respectively. As shown in Figure 3A, cell migration of esophageal cancer cells was significantly suppressed by siRNP2 treatment compared with siNC treatment (*P*<0.01). For CP-D cells, cell invasion rate was decreased to 23.41 ± 3.14% following siRNP2 transfection (Figure 3B). Similarly, cell invasion rate of CP-C cells transfected with siRPN2 was decreased to 18.67 ± 2.47% (Figure 3B). The results reveal that RPN2 knockdown might attenuate tumor metastasis in esophageal cancer.

### RPN2 regulated protein expressions of PCNA, MMP-2, E-cadherin, Snail and p-Smad2/3

**Figure 4:**
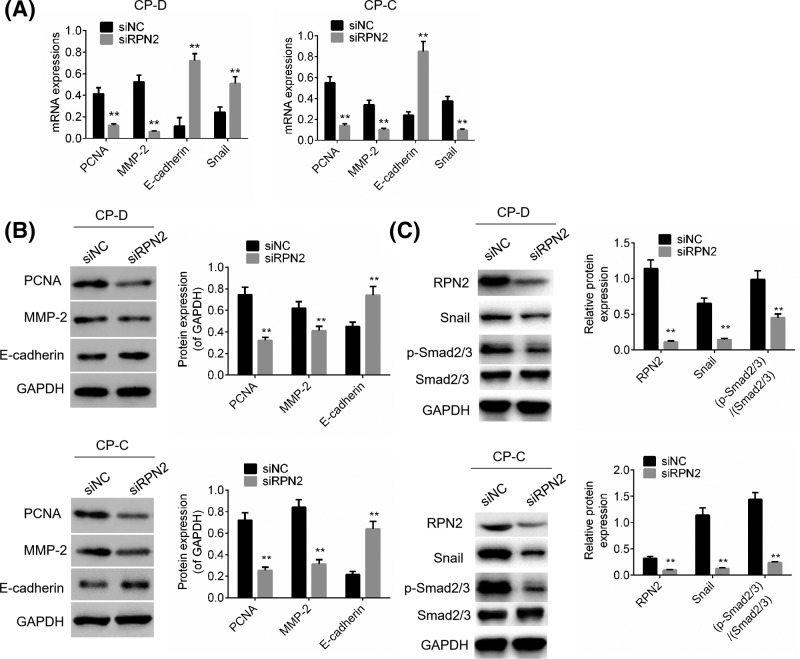
Effect of siRPN2 on PCNA, MMP-2, E-cadherin, Snail and p-Smad2/3 expression in esophageal cancer cells (**A**) The mRNA expressions of PCNA, MMP-2, Snail, and E-cadherin expression in CP-D and CP-C cells transfected with siRNP2 or siNC was detected by RT-PCR analysis. (**B**) PCNA, MMP-2, and E-cadherin expression in CP-D and CP-C cells transfected with siRNP2 or siNC was detected by Western blot analysis. (**C**) RPN2, Snail, p-Smad2/3 and Smad2/3 expression in CP-D and CP-C cells transfected with siRNP2 or siNC was detected by Western blot analysis. GAPDH was used as a loading control. Data are expressed as the mean ± S.D. for three independent experiments. ^**^P<0.01 versus siNC group.

To explore the underlying mechanism by which RPN2 regulates cell proliferation, migration, and invasion in esophageal cancer cells, protein expressions of PCNA, MMP-2, E-cadherin, and Snail were examined by RT-PCR and Western blot analysis. The p-Smad2/3/Smad2/3 was examined by Western blot. It was shown that down-regulation of RPN2 effectively inhibited PCNA, MMP-2, Snail and p-Smad2/3 expression in CP-D and CP-C cells (*P*<0.01, Figure 4A–C). Furthermore, E-cadherin expression in CP-D and CP-C cells transfected with siRPN2 were significantly increased compared with siNC transfected cells (*P*<0.01, Figure 4A,B). The results reveal that cell proliferation, migration, and invasion-related factors could be regulated by RPN2.

## Discussion

The incidence of esophageal cancer is increasing all over the world, and the development of tumor markers will be helpful for early diagnosis and improved prognosis of esophageal cancer. With the development of detection technology, tumor markers will become more and more important as auxiliary diagnostic means. RPN2 exerts tumor genetic functions in many kinds of cancer [15]. Honma *et al*. reported that RPN2 silencing induced apoptosis of breast cancer cells in the presence of docetaxel, *in vitro* and *in vivo* [16]. Fujita *et al*. reported that down-regulation of RPN2 significantly inhibited tumorigenicity and sensitized the tumors to cisplatin treatment, which lengthened the survival of mice with non-small-cell lung cancer [17]. Hong *et al*. demonstrated that siRNA-RPN2 could dramatically suppress the invasion and migration of human nasopharyngeal carcinoma cells through AKT/PI3K signaling [12]. Kurashige *et al.* reported that RPN2 expression in endoscopic biopsy specimens of oesophageal squamous cell carcinoma might predict response to docetaxel-based chemotherapy [14]. The role and mechanism of RNP2 in the progression of esophageal cancer was not investigated clearly. In our study, we report for the first time that RPN2 expression is significantly increased in the esophageal cancer tissue compared with normal tissue. In addition, RNP2 expression is also elevated notably in esophageal cancer cell lines compared with normal esophageal epithelial cell lines. These results indicate that RPN2 could exert an oncogenic role in esophageal cancer.

Abnormal expression of oncogene often gives rise to over-proliferation during the development and metastasis of esophageal carcinomas [18,19]. In the present study, we found that knockdown of RPN2 effectively inhibited cell proliferation of esophageal cancer cells. Down-regulation of RPN2 expression also inhibited tumor growth *in vivo*, and dramatically reduced tumor size and weight in a xeno-transplanted tumor model. Immoderate migration and invasion of tumor cells are considered crucial in the process of esophageal carcinoma metastasis [20,21]. Cell migration and invasion capabilities of esophageal cancer cells transfected with siRPN2 were then evaluated. Our results show that decreased expression of RPN2 significantly reduces cell migration and invasion of esophageal cancer cells. These results reveal that RPN2 could promote cell proliferation, migration, and invasion of esophageal cancer cells.

PCNA is closely related to DNA synthesis, plays an important role in the initiation of cell proliferation, and is a good indicator of cell proliferation. Increased PCNA expression is observed in a variety of tumors including esophageal carcinoma [22–24]. MMP-2 plays a crucial role in extracellular matrix degradation, which allows cancer cells to migrate out of the primary tumor to form metastases [25]. Down-regulation of E-cadherin leads to the decrease of intercellular adhesion junctions, the decrease of polarity and the transformation of cells from epithelioid to interstitial, which is one of the important markers of epithelial–mesenchymal transition (EMT) [26]. At the same time, EMT can promote the migration of tumor cells, enhance the invasion of tumor cells, and promote the occurrence of metastases [27,28]. E-cadherin has a certain correlation with the occurrence of many kinds of tumors. In the current study, the expression of PCNA, MMP-2, and E-cadherin was detected by Western blot analysis. We found that siRNA-RPN2 remarkably decreased the expression of PCNA and MMP-2, and significantly increased the expression of E-cadherin. RPN2 was reported to be related to the TGF-β downstream [9], and the expression of Snail and p-Smad2/3 was also evaluated. Down-regulation of RPN2 remarkably decreased the expression of Snail and p-Smad2/3. These findings demonstrate that RPN2 could regulate cell proliferation, migration, and invasion via accommodation of PCNA, MMP-2, and E-cadherin expressions, respectively.

In summary, we report that elevated RPN2 expression was seen in esophageal cancer and that RPN2 could promote cell proliferation, migration, and invasion of esophageal cancer cells. We also confirmed the anti-tumorigenic effects of silencing RPN2 expression in esophageal cells *in vivo*. Our study might provide novel ideas for esophageal cancer prevention and therapy. In addition, we found that Ribophorin I (RPN1) was also overexpressed in esophageal cancer samples, and exploring the effect of RPN1 on the occurrence and development of esophageal cancer cells and its molecular mechanism will be the focus of our work.

## Availability of data and materials

The datasets used and/or analyzed during the current study are available from the corresponding author on reasonable request.

## Supporting information

**Supplementary Figure 1:** The effect of RNP1 knockdown on cell proliferation and invasion of esophageal cancer cells. A. RPN1 and RPN2 expression in TCGA database. B. RPN1 expression in clinical esophageal cancer tissues and normal tissues by RT-PCR. C. CP-D and CP-C cells were transfected with siRPN1 plasmid, and RPN1 expression was identified by RT-PCR. D-E. Cell proliferation and invasion were examined by CCK8 and transwell assay. Data were shown as mean ± SD. ***P<0.001 vs siNC group.

## Abbreviations

CCK8: cell counting kit-8
EMT: epithelial–mesenchymal transition
H&E: hematoxylin and eosin
IHC: immunohistochemistry
MMP-2: matrix metalloproteinase
PBS: phosphate buffer saline
PCNA: proliferating cell nuclear antigen
qRT-PCR: quantitive Reverse transcription ploymerase chain reaction
RNP: ribophorin
TNM: tumor node metastasis
