# Expression of Concern: Ribophorin II promotes cell proliferation, migration, and invasion in esophageal cancer cells in vitro and in vivo

**DOI:** 10.1042/BSR-2018-2448_EOC

**Published:** 2024-03-01

**Keywords:** cell migration, cell proliferation, esophageal cancer, Invasion, RPN2

The Editorial Office has been made aware of potential issues surrounding the scientific validity of this paper, hence has issued an expression of concern to notify readers whilst the Editorial Office investigates. It has been noted that Figure 3A CP-C siNC 48 h panel seems to appear as Figure 7D shCtrl 48h panel in a paper by Yang et al 2020 (DOI: 10.18632/aging.103032).

