# Supplementary material for: Ribophorin II promotes cell proliferation, migration, and invasion in esophageal cancer cells in vitro and in vivo
Source: Biosci Rep. 2019 May 7;39(5):BSR20182448. doi: 10.1042/BSR20182448 (PMC6505194; doi:10.1042/BSR20182448)
Supplement: Supplementary file 1 [file bsr20182448_Supp1.pdf]

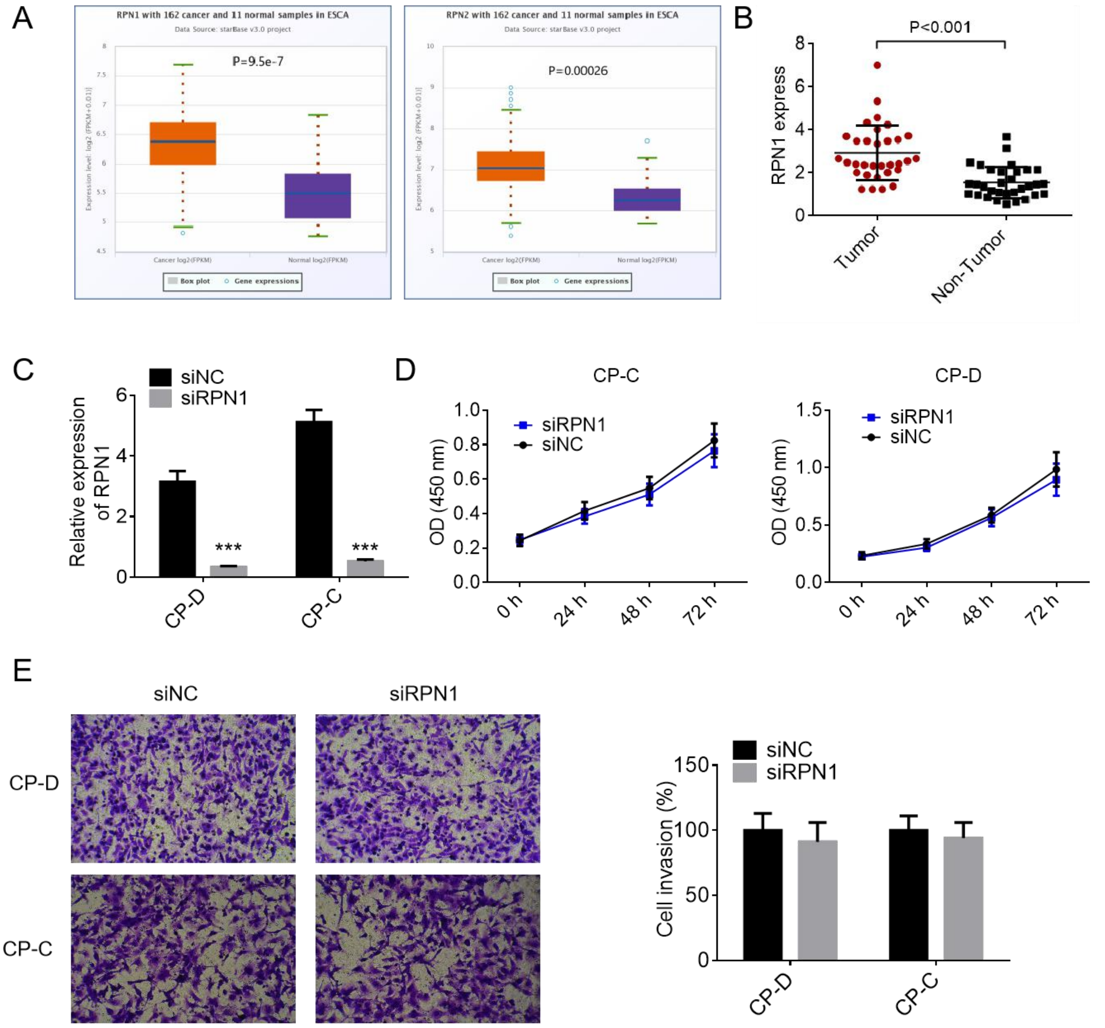

Supplementary figure 1. The effect of RNP1 knockdown on cell proliferation and invasion of esophageal cancer cells. A. RPN1 and RPN2 expression in TCGA database. B. RPN1 expression in clinical esophageal cancer tissues and normal tissues by RT-PCR. C. CP-D and CP-C cells were transfected with siRPN1 plasmid, and RPN1 expression was identified by RT-PCR. D-E. Cell proliferation and invasion were examined by CCK8 and transwell assay. Data were shown as mean ± SD. ***P<0.001 vs siNC group.
